# Impact of disease activity on health related quality of life in patients with Behçet's disease: A cross-sectional study

**DOI:** 10.1016/j.amsu.2020.03.010

**Published:** 2020-04-13

**Authors:** Faiq I. Gorial, Mais Ajeel Jabbar

**Affiliations:** a Department of Medicine, Collage of Medicine, University of Baghdad, Iraq; b Baghdad Teaching Hospital, Rheumatology Unit, Baghdad, Iraq

**Keywords:** Behcets disease, Disease activity, Health related quality of life

## Abstract

**Background:**

Behçet's syndrome (BD) is a systemic inflammatory vasculitis of unknown aetiology, affecting vessels of different types, sizes and locations and characterized by recurrent episodes of acute inflammation, including mucocutaneous manifestations (oral aphthous ulcers, genital ulcers and skin lesions) and gastrointestinal, musculoskeletal, neurological, ophthalmic and vascular involvement which lead to a significant morbidity and impaired health related quality of life (HRQoL). Few studies reported impact of disease activity on HRQoL.

**Objective:**

To assess the impact of BD activity on HRQoL.

**Patients and methods:**

This cross sectional study included patients with Behçet's disease diagnosed according to the International Study Group criteria 1990 for BD. Age of the patients, sex, smoking status, educational level, disease duration, organ involvement, age at disease onset, and medications used were recorded. Behçet's Disease Disease activity was assessed using Behçet's Disease Current Activity Form (BDCAF) and HRQoL was evaluated using The Short Form-36 (SF-36).

**Results:**

A total of 71 patients (45 males, 26 females) with Behçet's disease were enrolled in this study. Mean age of patients was 36.0 ± 10.8 years, Males represented the majority of patients (63.4%). BDCAF was significantly and negatively correlated with total SF-36 score (standardized β = − 0.520, p < 0.0001). The mean BDCAF was significantly more in females compared to males (6.154 ± 2.444 vs 4.467 ± 2.785, p = 0.012). While the mean SF36 was significantly more in males compared to females (57.722 ± 21.627 vs 41.435 ± 18.993, p = 0.002). After multiple linear stepwise regression analysis, still BDCAF significantly and negatively affected HRQoL in BD (partial r = −0.255, p = 0.043). Male gender, cyclosporine users, infliximab users, and Adalimumab users had significant positive impact on total SF-36 score (partial r = 0.293, p = 0.020; partial r = 0.256, p- = 0.043, partial r = 0.414; p = 0.00, partial r = 0.399, p = 0.001 respectively). While disease duration, and MMF users (partial r = −0.295, p = 0.019; partial r = −0.250, p = 0.043) had significant negative impact on total SF-36 score, and there was weak positive correlation between vascular involvement and total SF36 score (partial r = 0.244,p = 0.053) and a negative weak correlation between the use of anticoagulant with total SF-36 score (partial r = −0.233, p = 0.066).

**Conclusions:**

Behçet's disease activity has a significant negative impact on HRQoL This may suggest that treating activity of disease may improve HRQoL**.**

## Highlights

• Behcets disease activity has significantly negative impact on HQoL.
• This may suggest that treating activity of disease may improve HRQoL**.**

## 1 Introduction

Behçet's disease is a systemic autoinflammatory disease with a chronic, relapsing‐remitting course hallmarked predominantly by mucocutaneous lesions and ocular involvement [1]. The disease is most commonly distributed along the ancient silk route but has been reported from almost all continents [1,2]. The estimated prevalence of 1.7 BD patients for 10,000 Iraqi population is more or less similar to the prevalence in other Mediterranean and Far East countries, excluding Turkey [3]. The aetiology of BD is still unclear and human leukocyte antigen (HLA)-B51 is believed to be the strongest risk factor for BD [4]. Genetic, environmental, and immunological factors are suggested to be implicated in the development of BD [5]. Infectious agents lead to an immune response in genetically predisposed individuals and have long been proposed as triggering factors in BD development [6].

The World Health Organization (WHO) put forward a definition of Health-Related Quality of Life (HRQOL) In 1993: the perception by the individual regarding their position in life, in the context of their culture and value systems, and in relation to their goals, expectations, standards, and concerns [7]. The Short Form-36 (SF-36), the most widely used generic HR-QOL measure, was administered [8].

Chronic rheumatologic problems in patients with Behçet's disease are reported to limit their daily activities and have a negative impact on their self-esteem and relationships with others [9]. Most of the previous studies viewed the relationship between disease activity and Quality of Life (QoL) [10,11], compared the QoL of patients with other patients group [12] or assessed the specific impact of the type and number of symptoms on the QoL of BD patients [13]. However, Behçet's disease may negatively affect patients physically, mentally and socially and may decrease their QoL significantly (9). This study was designed to assess the impact of disease activity on HRQoL among Iraqi patients with BD.

## 2 Patients and methods

### 2.1 Study design and setting

This cross sectional study was conducted at the Rheumatology Unit of Baghdad Teaching Hospital in Medical City from July 2017 to January 2018. Informed consent was obtained from each participant included in this study according to the declaration of Helsinki. Ethical approval was obtained from the Ethics Committee in Medical Department, College of Medicine, University of Baghdad. This study has been done and reported in line with the STROCSS criteria [14].

### 2.2 Sample selection

A total of 71 consecutive patients (45 male: 26 female) were classified as BD by fulfilling the International Study Group criteria 1990 for BD [15]. Patients were excluded from the study if they had any of the following: Patient suspected to have BD clinically but did not fulfil the inclusion criteria; Pregnancy, chronic diseases, psychiatric disorders, cancer, and dependence on alcohol or other substances.

### 2.3 Data collection

Data were gathered using a pre-constructed data collection sheet for patients including age, sex, smoking status, disease duration, age at disease onset, and BD organ involvements were reported. Height in centimetres and weight in kilograms were measured for all patients, body mass index (BMI) was calculated according to the equation BMI = weight/height 2, disease activity and medications were recorded for all patients. A full history was taken, and a medical examination was performed for all included subjects, and the data were obtained by interviewing the patients on a one-on- one basis in a private room to make the patients feel more comfortable while answering the questions.

## 3 Measures

### 3.1 The Behçet's disease current activity form (BDCAF)

BDCAF [16] depends on accurate history of clinical features present during the month prior to the date of assessment. New clinical features present over the preceding 28 days were scored [17]. The BDCAF is easy to complete and a reliable method of assessing and documenting clinical activity in BD patients for use in routine clinical practice. It has a good inter observer reliability for general disease activity [18]. BDCAF was revised in 2006 and available at (www.behcetdiseasesociety.org/).

### 3.2 The Short Form-36 (SF-36)

The Short Form-36 (SF-36) is the most widely used health-related quality of life measure in research to date. SF-36 items and scales are scored so that a higher score indicates a better. There are currently two sources for the SF-36 and scoring instructions: licensing them from Optum, Inc., or obtaining them from publicly available documentation from the **R**esearch **AN**d **D**evelopment (RAND) Corporation [19].

The RAND-36 Item Health Survey 1.0 (distributed by RAND) includes the same items as those in the SF-36 that are distributed by **M**edical **O**utcomes **S**tudy (MOS) Trust, Inc. but the recommended scoring algorithm is somewhat different [20]. The RAND-36 and its scoring instructions are publicly available on the RAND Corporation web site [21].

RAND-36 assesses eight health concepts with multi-item scales (35 items): physical functioning (10 items), role limitations caused by physical health problems (4 items), role limitations caused by emotional problems (3 items), social functioning (2 items), emotional wellbeing (5 items), energy/fatigue (4 items), pain (2 items), and general health perceptions (5 items). An additional single item assesses change in perceived health during the last 12 months. Physical and mental health summary scores are also derived from the eight RAND-36 scales. The most common scoring approach for the RAND-36 items boils down to transforming every item linearly to a 0–100 possible range (percent of total possible score) and then averaging all items in the same scale together [22]. Total SF-36 score was calculated by averaging all items in all scales collectively.

### 3.3 Statistical analysis

Descriptive statistics were presented as mean ± SD for continuous variables with normal distributions and number (percentages) for categorical variables. Pearson correlation ecoefficient was used to assess the correlation between SF36 and BDCAF. Multiple linear stepwise regression was used to assess the impact of baseline characteristics on SF36. P < 0.05 was statistically significant. Statistical software SPSS v20 was used for analysis.

## 4 Results

A total of 71 patients (45 males, 26 females) with Behçet's disease were enrolled in this study. Mean age of the patients was 36.0 ± 10.8 years. Most of them were males 45 (63.4%). Mean Body mass index was 28.7 ± 6.1 kg/m2. Most of the patients 36 (50.7%were of primary educational level. Smokers were 20(28.2%) patients. The mean age at onset of the disease was 27.8 ± 10.3 years. While the mean disease duration since diagnosis was 5.1 ± 6.0 years and the mean of symptoms duration was 8.3 ± 7.1 years. The mean disease activity measured by BDCAF was 5.1 ± 2.8. All of the patients had mucosal involvement (100%), then ocular involvement was second in frequency 62 (87.3%), and thirdly was the articular involvement 51 (71.8%). Colchicin was the commonest medication used 41 (57.7%), next was infliximab 34 (47.9%), then steroids 32 (45.1%) as shown in Table 1, Table 2.

**Table 1:** Demographic characteristics of 71 BD patients.

Variables	value
Number	71
**Age (years), mean ± SD**	36.0 ± 10.8
**Gender, No. (%)**	
Female	26 (36.6%)
Male	45 (63.4%)
**BMI (kg/m**^**2**^**), mean ± SD**	28.7 ± 6.1
**Education level, no. (%)**	
Primary	36 (50.7%)
Intermediate	12 (16.9%)
Secondary	11 (15.5%)
College	11 (15.5%)
Post-graduate	1 (1.4%)
**Smoking, no. (%)**	20 (28.2%)
**Age at disease onset (years), mean ± SD**	27.8 ± 10.3
**Disease duration since diagnosis (years), mean ± SD**	5.1 ± 6.0
**Symptoms duration (years), mean ± SD**	8.3 ± 7.1
**BDCAF, mean ± SD**	5.1 ± 2.8

BD: Behçet's disease, BDCAF: Behçet's Disease Current Activity Form, n, number, %: percent, S: significant, SD: standard deviation.

**Table 2:** Clinical and treatment related parameters for BD patients.

Variables	Value
Organ involvements, No. (%)	
Mucosal	71 (100%)
Eye	62 (87.3%)
Articular	51 (71.8%)
Skin	48 (67.6%)
Myalgia	16 (22.5%)
CNS	9 (12.7%)
Vessels	3 (4.2%)
GIT	1 (1.4%)
**Treatments, No. (%)**	
Colchicine	41 (57.7%)
Infliximab	34 (47.9%)
Steroids	32 (45.1%)
Azathioprine	19 (26.8%)
Adalimumab	3 (4.2%)
Cyclosporine	2 (2.8%)
Sulphasalazine	2 (2.8%)
MTX	2 (2.8%)
Dapsone	2 (2.8%)
MMF	1 (1.4%)
Rituximab	1 (1.4%)
Anticoagulant	1 (1.4%)

Abbreviations: BD: Behçet's disease, CNS: Central nervous system, GIT: Gastrointestinal tract, MMF: Mycophenolate mofetil, MTX: methotrexate, No: Number %: percent.

**Fig. 1:**
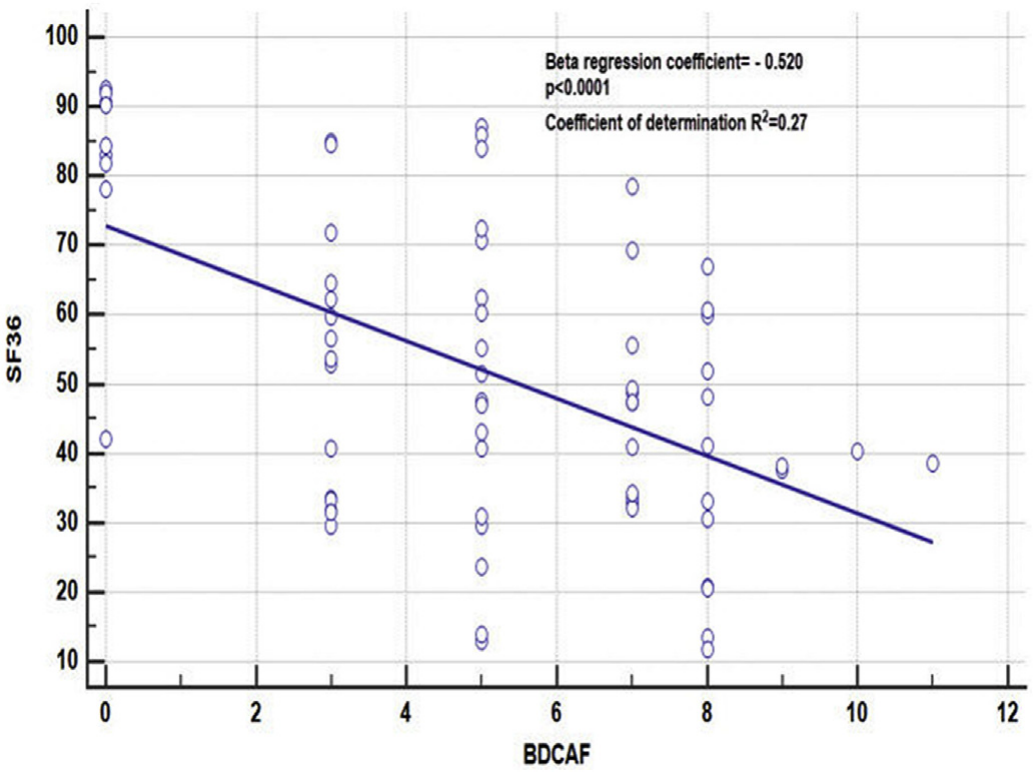
Correlation between Behcet disease current activity form (BDCAF) and short form 36 (SF36)

BDCAF was significantly and negatively correlated with total SF-36 score (standardized β = − 0.520, p < 0.0001) (Fig. 1).

The mean BDCAF was significantly more in females compared to males (6.154 ± 2.444 vs 4.467 ± 2.785, p = 0.012). While the mean SF36 was significantly more in males compared to females (57.722 ± 21.627 vs 41.435 ± 18.993, p = 0.002) as shown in Table 3.

**Table 3:** Activity of BD and HRQoL according to sex distribution.

Variable	Group	N	Mean	SD	P
BDCAF	female	26	6.154	2.444	0.012
	male	45	4.467	2.785	
SF36	female	26	41.435	18.993	0.002
	male	45	57.722	21.627	

BD, Behcets disease, HRQoL, health related quality of life; BDCAF, Behcets disease current activity form; SF36, short form 36.

After multiple linear stepwise regression analysis, still BDCAF significantly and negatively affected HRQoL in BD (partial r = −0.255, p = 0.043) as shown in Table 4.

**Table 4:** Multiple linear regression to predict the effect of baseline characteristics on SF36.

Variables	Partial r	p-value
Gender (male)^a^	0.293	0.020
Disease duration	−0.295	0.019
BDCAF	−0.255	0.043
Vessels ^a^	0.244	0.053
Cyclosporine ^a^	0.256	0.043
MMF ^a^	−0.250	0.048
Infliximab ^a^	0.414	0.001
Adalimumab ^a^	0.399	0.001
Anticoagulant ^a^	−0.233	0.066

Multiple linear stepwise regression using dummy variables and backward elimination method, Correlation coefficient (r) used to represent the magnitude of correlation.

Abbreviations: ^a^ Dummy variables; BDCAF, Behcet's Disease Current Activity Form; MMF, mycophenolate mofetil; P value, Probability Value (<0.05).

Male gender, cyclosporine users, infliximab users, and Adalimumab users had significant positive impact on total SF-36 score (partial r = 0.293, p = 0.020; partial r = 0.256, p- = 0.043, partial r = 0.414; p = 0.00, partial r = 0.399, p = 0.001 respectively). While disease duration, and MMF users (partial r = −0.295, p = 0.019; partial r = −0.250, p = 0.043) had significant negative impact on total SF-36 score, and there was weak positive correlation between vascular involvement and total SF36 score (partial r = 0.244,p = 0.053) and a negative weak correlation between the use of anticoagulant with total SF-36 score (partial r = −0.233, p = 0.066) as in Table 4.

## 5 Discussion

This study assessed the impact of BD activity on HRQoL and revealed that disease activity measured by BDCAF had a significant negative impact on HRQoL.

Previous studies have reported that the overall disease activity measured by BDCAF had significantly impaired HRQoL in all the SF-36 subscores in BD patients [23,24].

Similar finding was reported by Bodur et al. who investigated the association between HRQoL and disease activity measured by BDCAF and showed that active BD had a negative impact on HRQoL 10].

In the current study, the frequency of males was significantly more than that of females, in addition activity of BD disease was significantly more in females than males, however the HRQoL was more significantly in males compared to females. Similar finding was reported by Gheita et al. [25] who found that males were more than females The sex influences the disease phenotype with the neurological, vascular, and gastrointestinal involvement were higher in males, while the joint affection and BD disease activity were increased in females. However, in that study they did not assess HRQoL.

The current study showed that the use of cyclosporine, infliximab, and adalimumab was associated with better HRQoL among BD patients. While the use of mycophenolate mofetil (MMF) was associated with lower HRQoL among BD patients. Previous study [13] evaluated the effect of different treatment modalities on HRQoL of BD patients with ocular involvement and showed that patients who were taking conventional immunosuppressive agents had significantly lower general perception of health than those who were taking biologic agents. The main biologic agent used in that study population was interferon alfa-2α. Another study evaluated treatment modalities and quality of life in BD patients revealed that both oral HRQoL and Scores of SF-36 (Bodily pain, General health and Vitality) were significantly higher in patients using immunosuppressive compared with colchicine group as immunosuppressive treatments eliminate oral ulcers efficiently and suppress other disease activity [11]. In general the use of biologic agents and immunosuppressive medications were associated with better disease control and hence a higher HRQoL.

This study had some Limitations: the small sample size and short duration of the study led to inability to evaluate QoL in patients with large vessel and several systemic involvements. Also this study was cross sectional study and we can not assess the causality between the increase in BD activity and impaired HRQoL, however, up to the best of our knowledge, it is the first study that assessed the HRQoL among Iraqis.

## 6 Conclusion

Bechet's disease activity has significantly a negative impact on HRQoL This may suggests that treating activity of disease may improve HRQoL**.**

## Provenance and peer review

Not commissioned, externally peer reviewed.

## Ethical approval

The local scientific ethics committee of Department of Medicine, College of Medicine, University of Baghdad approved the study protocol.

## Sources of funding

No sources of funding.

## Author contribution

Both authors(Faiq I. Gorial and Mais Ajeel Jabbar) contributed in concept or design of the study, data collection, data analysis or interpretation, writing the paper, and approval of the final version of the paper.

## Consent

All patients signed written informed consent for participation in the study.

## Registration of research studies

The local scientific ethics committee of Department of Surgery, College of Medicine, University of Baghdad approved the study protocol with number 10 on November 15, 2018.

Research registry UIN 5301.

Link: https://www.researchregistry.com/browse-the-registry.

## Guarantor

Faiq I. Gorial.

## Declaration of competing interest

No Conflicts of interests.
